# Antimicrobial Activity against Foodborne Pathogens and Antioxidant Activity of Plant Leaves Traditionally Used as Food Packaging

**DOI:** 10.3390/foods12122409

**Published:** 2023-06-19

**Authors:** Wisuwat Thongphichai, Veerachai Pongkittiphan, Areerat Laorpaksa, Worakorn Wiwatcharakornkul, Suchada Sukrong

**Affiliations:** 1 Department of Pharmacognosy and Pharmaceutical Botany, Faculty of Pharmaceutical Sciences, Chulalongkorn University, Bangkok 10330, Thailand; 2 Center of Excellence in DNA Barcoding of Thai Medicinal Plants, Chulalongkorn University, Bangkok 10330, Thailand

**Keywords:** natural preservative, natural food packaging, antimicrobial activity, antioxidant, phenolics, bioassay-guided isolation

## Abstract

In accordance with Thai wisdom, indigenous plant leaves have been used as food packaging to preserve freshness. Many studies have demonstrated that both antioxidant and antimicrobial activities contribute to protecting food from spoilage. Hence, the ethanolic extracts of leaves from selected plants traditionally used as food packaging, including *Nelumbo nucifera* (**1**), *Cocos nucifera* (**2**), *Nypa fruticans* (**3**), *Nepenthes mirabilis* (**4**), *Dendrocalamus asper* (**5**), *Cephalostachyum pergracile* (**6**), *Musa balbisiana* (**7**), and *Piper sarmentosum* (**8**), were investigated to determine whether they have antioxidant and antimicrobial activities against spoilage microorganisms and foodborne pathogens that might be beneficial for food quality. Extracts **1**–**4** exhibited high phenolic content at 82.18–115.15 mg GAE/g and high antioxidant capacity on DPPH, FRAP and SRSA assay at 14.71–34.28 μg/mL, 342.92–551.38 μmol Fe^2+^/g, and 11.19–38.97 μg/mL, respectively, while leaf extracts **5**–**8** showed lower phenolic content at 34.43–50.08 mg GAE/g and lower antioxidant capacity on DPPH, FRAP, and SRSA at 46.70–142.16 μg/mL, 54.57–191.78 μmol Fe^2+^/g, and 69.05–>120 μg/mL, respectively. Extracts **1**–**4** possessed antimicrobial activities against food-relevant bacteria, including *Staphylococcus aureus, Bacillus cereus, Listeria monocytogenes,* and *Escherichia coli*. Only *N. mirabilis* extract (**4**) showed antimicrobial activities against *Salmonella enterica* subsp. *enterica* serovar Abony and *Candida albicans*. Extracts **5**–**8** showed slight antimicrobial activities against *B. cereus* and *E. coli*. As the growth and activity of microorganisms are the main cause of food spoilage, *N. fruticans* (**3**) was selected for bioassay-guided isolation to obtain 3-*O*-caffeoyl shikimic acid (**I**), isoorientin (**II**) and isovitexin (**III**), which are responsible for its antimicrobial activity against foodborne pathogens. *N. fruticans* was identified as a new source of natural antimicrobial compounds **I**–**III**, among which 3-*O*-caffeoyl shikimic acid was proven to show antimicrobial activity for the first time. These findings support the use of leaves for wrapping food and protecting food against oxidation and foodborne pathogens through their antioxidant and antimicrobial activities, respectively. Thus, leaves could be used as a natural packaging material and natural preservative.

## 1. Introduction

The main spoilage mechanisms of a great variety of food products, such as nuts, fish, meat, whole-milk powder, sauces, and oils, usually involve oxidation and microbial growth [1]. Food spoilage causes losses of both sensory and nutritional quality [2]. Foodborne pathogens, such as *Staphylococcus aureus*, *Bacillus cereus*, *Listeria monocytogenes*, *Escherichia coli*, *Salmonella* spp., and *Aspergillus* spp., could possibly contaminate and develop during food production, processing, storage, and transportation [3]. Some bacteria and fungi also produce toxins, leading to chemical and biological food poisoning outbreaks [4]. To prevent food spoilage, some synthetic antioxidants, such as butylhydroxyanisole (BHA) and butylhydroxytoluene (BHT), have been added to foods. However, because of the potential health hazards of these chemical additives, there have many attempts to seek alternative food packaging materials with antioxidant and antibacterial activities that extend the shelf life of food without the addition of chemical additives [5].

**Figure 1:**
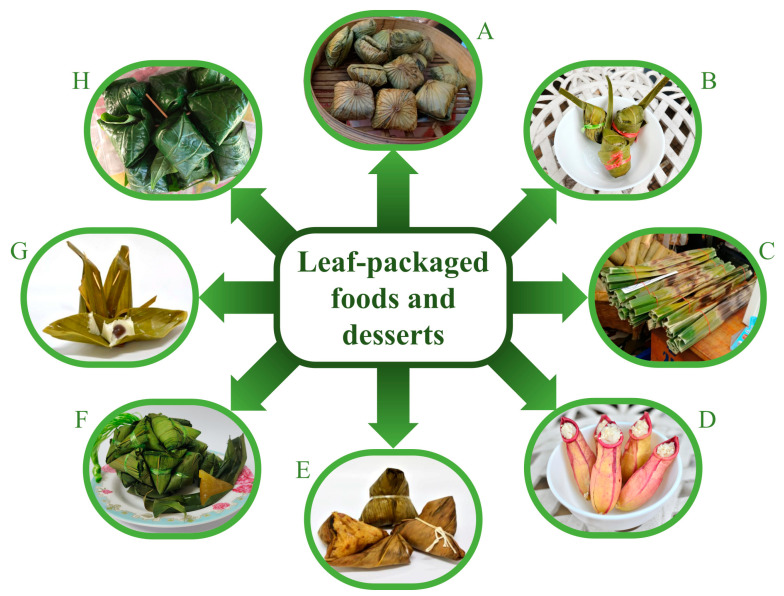
Traditional foods and desserts wrapped with leaves from plants, including *N. nucifera* (**A**), *C. nucifera* (**B**), *N. fruticans* (**C**), *N. mirabilis* (**D**), *C. pergracile* (**E**), *D. asper* (**F**), *M. balbisiana* (**G**), and *P. sarmentosum* (**H**).

In Asian countries, leaves from local plants have been used as packaging for foods or desserts (Figure 1A–H) to ensure safe food handling and facilitate convenient consumption. Their waxy and waterproof surfaces protect food from excessive moisture and retard the spoilage process [6]. Leaf-packaged food also has an attractive shape and preserved freshness. Previous studies have demonstrated that some leaf extracts have antioxidant and antimicrobial activities that play an important role in food spoilage prevention [7,8]. In Thailand, several plant leaves have been used as food packaging. However, there are no studies that demonstrate both antioxidant and antimicrobial activities against spoilage organisms and foodborne pathogens of plant leaves traditionally used as food packaging. Herein, leaves from selected plants, namely sacred lotus (*Nelumbo nucifera* Gaertn.; **1**)*,* coconut (*Cocos nucifera* L.; **2**), nipa palm (*Nypa fruticans* Wurmb; **3**), swamp pitcher plant (*Nepenthes mirabilis* (Lour.) Druce; **4**), giant bamboo (*Dendrocalamus asper* (Schult & Schult.f.) Backer; **5**), tinwa bamboo (*Cephalostachyum pergracile* Munro; **6**), banana (*Musa balbisiana* Colla; **7**), and wild betal (*Piper sarmentosum* Roxb.; **8**), were subjected to testing for their antioxidant activity. They were also subjected to antimicrobial activity testing against selected foodborne pathogens, including Gram-positive bacteria (*S. aureus, B. cereus,* and *L. monocytogenes*), Gram-negative bacteria (*E. coli* and *Salmonella enterica* subsp. *enterica* serovar Abony (*Salmonella* Abony)), and fungi (*Candida albicans* and *Aspergillus niger*), to evaluate and compare the bioactivity of these plant leaves. In addition, the active compounds responsible for the antimicrobial activity of frequently used leaves, *N. fruticans*, were isolated using bioassay-guided isolation and subsequently elucidated for chemical structures.

## 2. Materials and Methods

### 2.1. Chemicals

High-performance thin-layer chromatography (HPTLC) silica gel 60 F_254_ glass plates, 2,2-diphenyl-1-picrylhydrazyl (DPPH), Folin–Ciocalteu (FC) reagent, gallic acid, ascorbic acid, hydrochloric acid (HCl), formic acid, reagent grade butanol (BuOH), absolute ethanol (EtOH), diphenylborinic acid aminoethyl ester, polyethylene glycol 400, 2,4,6-tri(2-pyridyl)-*s*-triazine (TPTZ), iron (III) chloride hexahydrate (FeCl_3_·6H_2_O), iron (II) sulfate heptahydrate (FeSO_4_·7H_2_O), disodium ethylenediaminetetraacetate (Na_2_-EDTA), sodium carbonate (Na_2_CO_3_), nitroblue tetrazolium (NBT), riboflavin, Trolox, quercetin, gentamicin, and amphotericin B were purchased from Sigma-Aldrich (Burlington, MA, USA) and Merck KGaA (Darmstadt, Germany). Mueller–Hinton Broth, Mueller–Hinton agar, and Sabouraud dextrose agar were purchased from Difco (BD Diagnostics, Sparks, MD, USA). DMSO-d6 was acquired from Cambridge Isotope Laboratories, Inc. (Tewksbury, MA, USA). Deionized water was generated with a Barnstead™ MicroPure™ Water Purification System (Thermo Fisher Scientific, Waltham, MA, USA). Commercial-grade hexanes, dichloromethane, ethyl acetate (EtOAc), methanol (MeOH), and 95% EtOH were acquired from T.S. Interlab LP (Bangkok, Thailand) and distilled before use.

### 2.2. Plant Collection and Extraction

Leaves of *N. nucifera* (**1**), and *N. mirabilis* (**4**) were collected from Queen Sirikit Botanic Garden, Chiang Mai, Thailand. *C. nucifera* (**2**), *N. fruticans* (**3**), *D. asper* (**5**), and *C. pergracile* (**6**) leaves were collected from Lung Choke Garden, Nakhon Ratchasima, Thailand. *M. balbisiana* (**7**) and *P. sarmentosum* (**8**) leaves were collected from the Medicinal Plant Garden, Chulalongkorn University. The taxonomic identification of plants was confirmed by Assoc. Prof. Thatree Phadungcharoen, a botanist at Chulalongkorn University. The voucher specimens were deposited at the Herbarium of Natural Medicines, Faculty of Pharmaceutical Sciences, Chulalongkorn University, Bangkok, Thailand (Table 1). The plant leaf materials were dried at 50℃ overnight, ground into small pieces, and successively extracted by maceration in 95% ethanol until exhausted. The leaf extracts were filtered and evaporated using a rotary evaporator to obtain ethanolic extracts **1**–**8**.

### 2.3. HPTLC Analysis and HPTLC–DPPH Bioautography of Leaf Extracts

Each leaf extract was dissolved in EtOH to afford a concentration of 10 mg/mL. Ten microliters of extracts **1**–**8** were spotted on HPTLC glass plates (20 cm × 10 cm) using an HPTLC applicator (CAMAG, Muttenz, Switzerland) with a 6 mm band width. The starting position was 15 mm from the edge and 10 mm from the bottom of the plate. The HPTLC plates were developed using a mixture of EtOAc–MeOH–formic acid (9:1:1 *v*/*v*) in a developing chamber. To visualize flavonoids and vegetable acids in the extracts, a developed HPTLC plate was derivatized with a natural product (NP) reagent (a mixture of 1 g diphenylborinic acid aminoethyl ester in 200 mL of EtOAc and 1 g PEG 400 in 20 mL of dichloromethane) and dried with warm air for 5 min. Then, the derivatized plates were observed under UV light at 365 nm.

TLC bioautography based on the DPPH assay was carried out to observe the antioxidant compounds. A developed HPTLC plate of leaf extracts was sprayed with DPPH solution (10 mM in EtOH) and kept in the dark for 5 min. The antioxidant components in the leaf extracts were observed as yellow spots.

### 2.4. Total Phenolic Content Assay

The total phenolic content of the extracts was determined using the Folin–Ciocalteu (FC) assay [9], with some modifications. The FC reagent was used at tenfold dilution in water. Briefly, 20 μL of test extracts (1.0 mg/mL in ethanol) and 100 μL of FC reagent were added together in a 96-well microplate, and then 80 μL of 7.5% (*w*/*v*) Na_2_CO_3_ solution was added. The microplate was incubated at room temperature for 30 min with occasional shaking. The absorbance was measured at 765 nm using a microplate reader. The absorbance values of several concentrations of gallic acid (20–160 μg/mL) were plotted as a standard curve to identify the total phenolic content of the leaf extracts. The results were presented as milligrams of gallic acid equivalent (GAE) per gram of dried extract. The assay was performed in triplicate.

### 2.5. Antioxidant Activity Assays

#### 2.5.1. DPPH Radical Scavenging Assay of Leaf Extracts

The DPPH radical scavenging assay used to assess the antioxidant capacity of leaf extracts [10] was performed with some modifications. Briefly, 50 μL of the leaf extract in ethanol (EtOH) at various concentrations (20–400 μg/mL) was added to 100 μL of 0.1 mM DPPH solution in a 96-well microplate. The microplate was incubated for 30 min in the dark at room temperature. The absorbance was measured at 510 nm using a Victor 3 multilabel plate reader (PerkinElmer, Waltham, MA, USA). EtOH and ascorbic acid were used as a blank and positive control, respectively. The DPPH radical scavenging activity was determined using the following formula:

$$\mathrm{DPPH}\;\mathrm{radical}\;\mathrm{scavenging}\;\mathrm{activity}\;\left(\%\right)=\frac{A_{c}-A_{s}}{A_{c}}\times 100$$

where A_c_ is the absorbance of DPPH without sample, and A_s_ is the absorbance of the samples mixed with DPPH solution. The assay was performed in triplicate.

#### 2.5.2. Ferric Reducing Antioxidant Power Assay

The ferric reducing antioxidant power (FRAP) assay was performed to identify the reducing ability of the leaf extracts [11]. The FRAP reagent was freshly prepared by mixing 300 mM acetate buffer (pH 3.6), 10 mM TPTZ in 40 mM HCl, and 20 mM FeCl_3_·6H_2_O, at a ratio of 10:1:1. In a 96-well microplate, 10 μL of each leaf extract sample (0.5 mg/mL) and 190 μL of FRAP reagent were mixed together and incubated at 37 °C for 30 min in the dark. The absorbance was measured at 595 nm using a microplate reader. The absorbance values of FeSO_4_·7H_2_O standard solutions (100–1400 μM) were plotted as a standard curve for the determination of ferric reducing capacity. The results are presented as mean ± SD (*n* = 3) of micromoles (μmol) of Fe^2+^ per gram of dried extract. The assay was performed in triplicate.

#### 2.5.3. Superoxide Radical Scavenging Assay

Formazan generation was measured in terms of the reduction in nitro blue tetrazolium (NBT) via the scavenging of superoxide radicals from a riboflavin–light–NBT system [12]. A mixture of 20 μL of a leaf extract sample, 100 μL of 50 mM phosphate buffer, 40 μL of 1 mM EDTA in phosphate buffer, 20 μL of 0.75 mM NBT in phosphate buffer, and 20 μL of 226 μM riboflavin in phosphate buffer, was added in a 96-well microplate. The reaction was induced via illumination with a 5W LED warm lamp (15 cm height from plate level) for 5 min. The absorbance was measured at 595 nm using Trolox and quercetin as positive controls. The inhibition of superoxide formation was calculated using the following equation:

$$\mathrm{Superoxide}\;\mathrm{radical}\;\mathrm{scavenging}\;\mathrm{activity}\;\left(\%\right)=\frac{A_{c}-A_{s}}{A_{c}}\times 100$$

where A_c_ is the absorbance of the control, and A_s_ is the absorbance of the leaf extract samples or standards. The assay was carried out in triplicate.

### 2.6. Antimicrobial Assay against Foodborne Pathogens

All the leaf extracts were dissolved in DMSO. Each extract solution was dropped onto a 6.0 mm Whatman paper disc at 10 mg of extract per disc, the maximum solubility of all extracts, and then all discs were dried in a laminar flow cabinet. Gentamicin (10 µg/disc) and amphotericin B (10 µg/disc) were used as positive controls. The cell suspensions of five foodborne bacteria, *S. aureus* ATCC 25923, *B. cereus* ATCC 11778, *L. monocytogenes* ATCC 7644, *E. coli* ATCC 25922, and *Salmonella* Abony NCTC 6017, were prepared to 0.5 McFarland turbidity standard (1.5 × 10^8^ CFU/mL). The fungal suspensions of *C. albicans* ATCC 10231 and *A. niger* ATCC 16404 were adjusted to a concentration of 1.5 × 10^6^ CFU/mL [13]. Twenty milliliters of Mueller–Hinton agar (MHA) and Sabouraud dextrose agar (SDA) were added into Petri dishes (9 cm diameter) for bacterial and fungal tests, respectively. Each pathogenic suspension was spread over the surface of an agar plate with a sterile cotton swab. The tested discs were placed on the spread plates and left for prediffusion for 1 h. The bacterial plates were incubated at 37 °C for 18–24 h, whereas fungal plates were incubated at 30 °C for 1–3 days. After the incubation period, the inhibition zone diameter in millimeters was measured using a Vernier caliper. The assays were performed in triplicate.

### 2.7. Bioassay-Guided Isolation of N. fruticans Extract to Obtain Antimicrobial Components

The ethanolic extract of *N. fruticans* (20 g) was suspended in a mixture of water and MeOH (7:3) and sonicated for 1 h at room temperature. The mixture was partitioned with 250 mL of hexanes, dichloromethane, EtOAc, and BuOH to provide a hexane fraction (**F1**), dichloromethane fraction (**F2**), EtOAc fraction (**F3**), BuOH fraction (**F4**) and water fraction (**F5**). Fractions **F1**–**F5** were evaporated to dryness and tested for antimicrobial activity against foodborne pathogens in comparison to *N. fruticans* extract using the disc diffusion method. The fraction with the most effective antimicrobial activity against foodborne pathogens was further separated via column chromatography to obtain pure compounds. Each isolated compound was identified via nuclear magnetic resonance spectroscopy and compared with previous reports.

### 2.8. Evaluation of Antimicrobial Activity of Compounds ***I**–**III*** against Foodborne Pathogens

The antimicrobial efficacy of compounds **I**–**III** was investigated using the dilution method with test tubes [14]. Isolated compounds **I**–**III** were dissolved in a 20% DMSO–water solution. Five foodborne pathogens, namely *S. aureus*, *B. cereus*, *L. monocytogenes*, *E. coli*, and *Salmonella* Abony, were prepared to 0.5 McFarland turbidity standard (1.5 × 10^8^ CFU/mL) in Mueller–Hinton Broth. The test solutions (0.5 mL) were added to pathogenic suspensions (0.5 mL) in a test tube. The final concentrations of isolated compounds were 1000, 800, 500, 400, 250, 125, 100, 62.5, 31.3, and 15.1 µg/mL. Gentamicin in phosphate buffer (pH 4.5) was used as a positive control. The final concentrations of gentamicin after mixing with each pathogenic suspension were 15.17, 7.59 3.79, 1.90, 0.95, 0.47, 0.24, 0.12, and 0.059 µg/mL. DMSO was used as a negative control. All test tubes were incubated at 37 °C for 18–24 h, and the turbidity was checked to determine the minimum inhibitory concentration (MIC) value.

### 2.9. Statistical Analysis

The data are presented as the means of three replicates ± standard deviations (SDs). The results were subjected to analysis of variance (ANOVA), and mean comparisons were performed with Tukey’s honestly significant difference test using GraphPad Prism 9 software. Differences between means were considered significant at a *p*-value < 0.05.

## 3. Results

### 3.1. HPTLC Profiles and HPTLC–DPPH Bioautograms of Leaf Extracts

**Figure 2:**
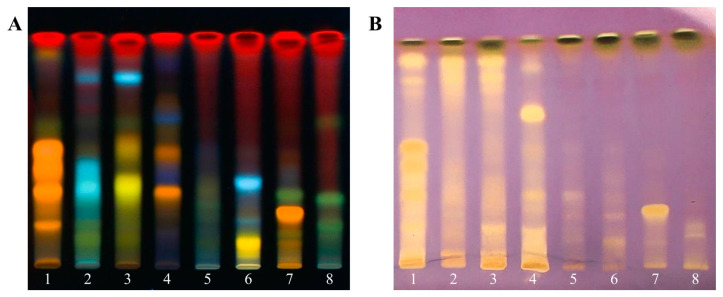
HPTLC chromatograms of *N. nucifera* (**1**), *C. nucifera* (**2**), *N. fruticans* (**3**), *N. mirabilis* (**4**), *C. pergracile* (**5**), *D. asper* (**6**), *M. balbisiana* (**7**), and *P. sarmentosum* (**8**) using ethyl acetate–methanol–formic acid (9:1:1 *v*/*v*) as mobile phase after spraying with NP reagent under UV 365 nm (**A**) and after development with DPPH reagent spray (**B**).

Leaves of all plant samples (Figure 1) were macerated with 95% EtOH until exhausted to obtain ethanolic extracts (Table 1). The greatest extraction yield of 39.32% was observed for *N. nucifera*, followed by extraction yields of 19.91, 17.47, 17.08, 14.15, 11.41, 7.93, and 5.20% for *C. nucifera*, *N. mirabilis*, *P. sarmentosum*, *N. fruticans*, *D. asper*, *C. pergracile*, and *M. balbisiana*, respectively. All extracts were analyzed using HPTLC. Each HPTLC plate was developed and sprayed with DPPH and NP reagents (Figure 2). Phytochemical screening by spraying NP reagent on an HPTLC plate disclosed a variety of fluorescence spots under UV 365 nm (Figure 2A). Extracts **1**, **3**, **4**, **6**, and **7** showed yellow and orange bands, while extracts **2**, **3**, and **6** showed light blue spots. However, the fluorescence band was absent for extract **5**. In addition, extracts **1**–**4** revealed a greater number of fluorescence spots than extracts **5**–**8**. To observe antioxidant compounds, an HPTLC plate was sprayed with the DPPH reagent (Figure 2B). Extracts **1**–**4** revealed more intense yellow spots than extracts **5**–**8**. The HPTLC results showed that extracts **1**–**4** contained many spots of chemical constituents with antioxidant activity. 

### 3.2. Total Phenolic Content and Antioxidant Activities of Leaf Extracts

All the leaf extracts were tested for total phenolic content and antioxidant capacity using DPPH radical scavenging, ferric reducing antioxidant power (FRAP), and superoxide radical scavenging (SRSA) assays (Table 2). The highest total phenolic content of 115.15 ± 3.84 mg GAE/g was observed for the *N. nucifera* extract (**1**), followed by *N. mirabilis* (**2**) (108.39 ± 6.48 mg GAE/g); *N. fruticans* (**3**) (97.54 ± 2.06 mg GAE/g); and *C. nucifera* (**4**) (82.18 ± 4.03 mg GAE/g) extracts. The other extracts **5**–**8**, namely *D. asper*, *C. pergracile*, *M. balbisiana*, and *P. sarmentosum*, showed lower total phenolic contents, with values ranging from 34.43 ± 0.27 to 50.08 ± 2.32 mg GAE/g. 

From the DPPH radical scavenging assay, the *N. nucifera* extract presented the greatest DPPH scavenging activity among all extracts, with an IC_50_ value of 14.71 ± 0.56 μg/mL, followed by *N. fruticans*, *N. mirabilis*, *C. nucifera*, *M. balbisiana*, *C. pergracile*, *P. sarmentosum*, and *D. asper*, with IC_50_ values of 14.93 ± 0.40, 16.67 ± 0.55, 34.28 ± 0.55, 46.70 ± 1.08, 61.69 ± 2.45, 116.09 ± 2.89, and 142.16 ± 3.25 μg/mL, respectively.

According to the FRAP assay, all extracts showed antioxidant efficacy, which was in agreement with the DPPH results. The highest ferric reducing antioxidant power was observed from the *N. mirabilis* extract with a value of 551.38 ± 4.11 μmol Fe^2+^/g, followed by the *N. nucifera*, *N. fruticans*, and *C. nucifera* extracts with values of 545.72 ± 10.80, 529.36 ± 5.44, 342.92 ± 8.51 μmol Fe^2+^/g, respectively. The other leaf extracts (*M. balbisiana*, *C. pergracile*, *P. sarmentosum*, and *D. asper*) showed lower FRAP activities (<200 μmol Fe^2+^/g). 

In the superoxide radical scavenging assay, the *N. nucifera* extract possessed the highest superoxide radical scavenging activity, with an IC_50_ value of 11.19 ± 0.63 μg/mL, followed by the *N. mirabilis, N. fruticans, C. nucifera*, and *P. sarmentosum* extracts with IC_50_ values of 20.16 ± 1.43, 27.89 ± 1.84, 38.97 ± 1.05, and 69.05 ± 1.5 μg/mL, respectively. According to the results, the *D. asper*, *C. pergracile*, and *M. balbisiana* extracts were observed to have the lowest scavenging activities among the test extracts, with IC_50_ values greater than 120 μg/mL. The results showed that extracts **1**–**4** contained higher phenolic content and antioxidant activity than extracts **5**–**8** in all assays. 

### 3.3. Antimicrobial Activity of Leaf Extracts

**Figure 3:**
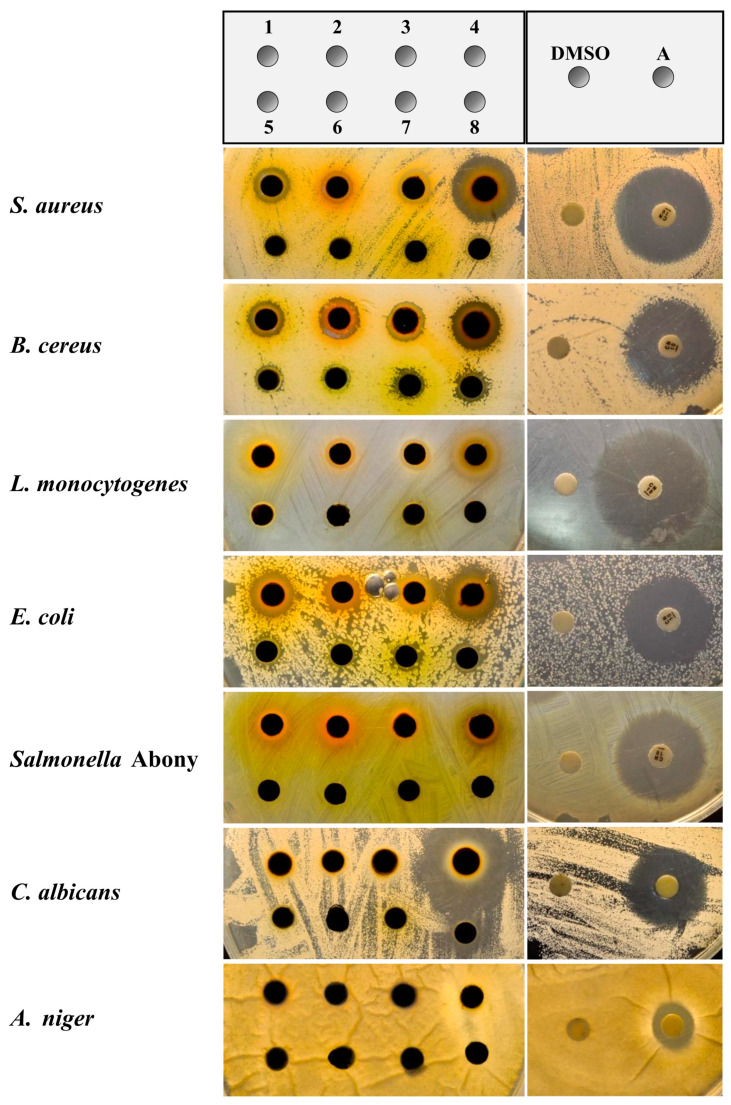
Inhibition zones of *N. nucifera* (**1**), *C. nucifera* (**2**), *N. fruticans* (**3**), *N. mirabilis* (**4**), *C. pergracile* (**5**), *D. asper* (**6**), *M. balbisiana* (**7**), and *P. sarmentosum* (**8**) extracts (10 mg/disc) against *S. aureus*, *B. cereus*, *L. monocytogenes*, *E. coli*, Salmonella Abony, *C. albicans*, and *A. niger*. DMSO was used as a control. A: antibiotic and antifungal, which was gentamycin and amphotericin B, respectively, at 10 µg/disc.

The antimicrobial activities of leaf extracts were examined by measuring the diameters of the inhibition zones of Gram-positive bacteria, namely *S. aureus*, *B. cereus*, and *L. monocytogenes*, and Gram-negative bacteria, namely *E. coli* and *Salmonella* Abony (Figure 3). The fungi *C. albicans* and *A. niger* were included in the test (Figure 3). Among all the extracts, *N. nucifera*, *C. nucifera*, *N. fruticans*, and *N. mirabilis* extracts exhibited antimicrobial activity against various types of bacteria, namely *S. aureus*, *B. cereus*, *L. monocytogenes*, and *E. coli.*

Notably, *N. mirabilis* extract showed the greatest activity against *S. aureus*, *B. cereus*, and *L. monocytogenes* with 17.77 ± 0.15, 13.10 ± 0.70, and 10.93 ± 0.61 mm inhibition zones, respectively (Table 3). *N. mirabilis* extract was the only extract that exhibited antimicrobial activity against *Salmonella* Abony and *C. albicans*, with 8.70 ± 0.53 and 24.10 ± 0.46 mm inhibition zones, respectively. *D. asper* displayed the greatest microbiostatic activity against *E. coli*, with a 14.67 ± 0.21 mm inhibition zone, and also showed a 7.80 ± 0.40 mm inhibition zone against *B. cereus*. However, the *D. asper* extract showed no inhibition zone against the other tested microbes. *C. pergracile*, *M. balbisiana*, and *P. sarmentosum* extracts displayed antimicrobial activity against only *E. coli*, with smaller inhibition zones when compared with the others. The results showed that extracts **1**–**4** exhibited antimicrobial activity against *S. aureus, B. cereus, L. monocytogenes*, and *E. coli*. Extract **4** also showed antimicrobial activity against *Salmonella* Abony and *C. albicans*. 

### 3.4. Bioassay-Guided Isolation of N. fruticans Leaf Extract 

**Figure 4:**
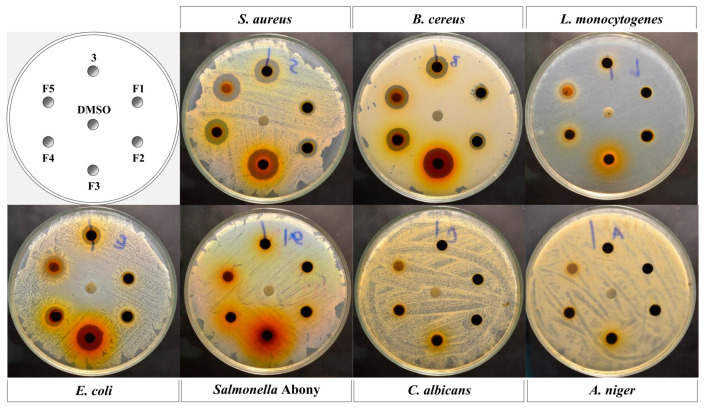
Inhibition zones of *N. fruticans* extract (**3**) and its different solvent fractions (10 mg/disc) against *S. aureus*, *B. cereus*, *L. monocytogenes*, *E. coli*, *Salmonella* Abony, *C. albicans*, and *A. niger*. DMSO was used as a vehicle control. **F1**: hexanes fraction; **F2**: dichloromethane fraction; **F3**: EtOAc fraction; **F4**: BuOH fraction; **F5**: water fraction.

**Figure 5:**
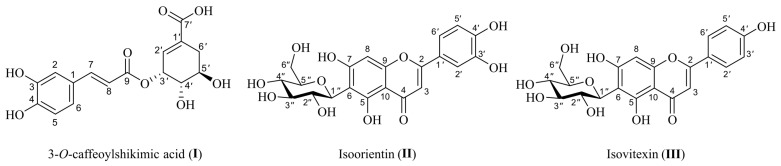
Structures of chemical constituents isolated from *N. fruticans;* 3-*O*-caffeoyl shikimic acid (**I**), isoorientin (**II**), and isovitexin (**III**).

The ethanolic extract of *N. fruticans* (**3**) was further partitioned with hexanes, dichloromethane, EtOAc, BuOH, and water, to afford five fractions. The extraction yields of hexanes (**F1**), dichloromethane (**F2**), EtOAc (**F3**), BuOH (**F4**), and water (**F5**) fractions were 22.3, 15.9, 4.1, 7.3, and 31.4%, respectively. All fractions were tested for antimicrobial activity against foodborne pathogens using the disc diffusion method (Figure 4). The results revealed that fraction **F1** had antimicrobial activity against *S. aureus*, *B. cereus*, and *L. monocytogenes*, with inhibition zone diameters of 8.93 ± 0.35, 8.27 ± 0.12 and 8.53 ± 0.15 mm, respectively (Table 4). Fractions **F4** and **F5** showed larger inhibition zones against *S. aureus*, *B. cereus*, *L. monocytogenes*, and *E. coli* than that of fraction **F1**, with the inhibition zone diameters varying from 7.83 ± 0.85 to 15.67 ± 0.86 mm. However, the inhibition zones of fractions **F1** and **F2** against *S. aureus*, *B. cereus*, and *L. monocytogenes* were significantly indifferent. Among all the tested fractions, fraction **F3** was the most active fraction against all microbes in the test. Fraction **F3** was purified using a Sephadex LH-20 column (MeOH was used as a mobile phase). Subfractions were further purified via column chromatography using a reversed-phase C-18 column, eluted with MeOH–water (60:40) as a mobile phase to obtain the compounds, which were characterized as 3-*O*-caffeoyl shikimic acid (**I**), isoorientin (**II**), and isovitexin (**III**) (Figure 5). 

Compound **I** was obtained as a bright yellow amorphous powder and showed a bright blue spot (Rf = 0.56) on a reversed-phase TLC plate under 365 nm UV light (mobile phase: 50% MeOH in water). Compound **I** was confirmed to be 3-*O*-caffeoyl shikimic acid by comparing ^1^H and ^13^C NMR spectroscopic data with those previously reported (Figures S1 and, S2 and Table S1). 

Compound **II** was obtained as a yellow amorphous powder and appeared as a yellow spot (Rf = 0.47) on a reversed-phase TLC plate under 365 nm UV light (mobile phase: 50% MeOH in water). Compound **II** was proven to be isoorientin by comparing ^1^H and ^13^C NMR spectroscopic data with those previously reported (Figures S3–S5 and Table S2). 

Compound **III** was isolated as a yellow amorphous powder and showed displaying an orange spot (Rf = 0.33) on a reversed-phase TLC plate under 365 nm UV light (mobile phase: 50% MeOH in water). Compound **III** was identified as isovitexin by comparing ^1^H and ^13^C NMR spectroscopic data with those previously reported (Figures S6 and S7 and Table S3).

### 3.5. Evaluation of the Antimicrobial Activity of Isolated Compounds ***I**–**III***

To test whether the isolated compounds had antibacterial activity, the MIC values of 3-*O*-caffeoyl shikimic acid (**I**), isoorientin (**II**), and isovitexin (**III**) (Table 5) against *S. aureus*, *B. cereus*, *L. monocytogenes*, and *E. coli* were evaluated using the tube dilution method. Based on our observations, *S. aureus* was less susceptible to **III** than **I** and **II**, with a MIC value greater than 1000 µg/mL. Compounds **I** and **III** exhibited greater antimicrobial activity against *B. cereus*, with a MIC value of 800 µg/mL, in contrast with that of 1000 µg/mL of **II**. *E. coli* was more vulnerable to **III** with a MIC value of 800 µg/mL, compared with that of 1000 µg/mL of **I** and **II.** Compound **III** displayed the greatest antimicrobial activity against *E. coli*, with a MIC value of 800 µg/mL, but showed the least efficacy against *Salmonella* Abony, with a MIC value greater than 1000 µg/mL. *L. monocytogenes* was equally vulnerable to all the tested compounds with a MIC value of 1000 µg/mL. The results showed that isolated compounds exhibited antimicrobial activity against *S. aureus*, *B. cereus*, *L. monocytogenes*, *E. coli*, and *Salmonella* Abony. 

## 4. Discussion

Selected plant leaves, namely *N. nucifera* (**1**), *C. nucifera* (**2**), *N. fruticans* (**3**), *N. mirabilis* (**4**), *D. asper* (**5**), *C. pergracile* (**6**), *M. balbisiana* (**7**), and *P. sarmentosum* (**8**), which have been used to wrap food (Figure 1A–H) in Thai ethnic culture, were studied for their beneficial as natural food preservatives. Interestingly, our selected plants have also been used in other countries. A Chinese rice pudding, *zongzi*, is wrapped with *N. nucifera* or *C. nucifera* leaves [15]. *N. fruticans* leaves are popularly used for wrapping desserts called *khanom chak* in Thailand (Figure 1C) and a type of rice cake, *ketupat*, in Malaysia [16]. Bamboo leaves, such as *D. asper* and *C. pergracile*, are used for wrapping streamed rice cakes in Japan [16]. *N. mirabilis* is an exotic plant, and its leaves develop into pitchers in order to trap insects. The pitchers of *N. mirabilis* are used as containers for a rare traditional dessert found in the southern part of Thailand (Figure 1D) [17]. Banana leaves, *M. balbisiana*, are used for wrapping a grilled fish fillet in India [18] and traditional desserts in Thailand (Figure 1G). *P. sarmentosum* leaves are used for wrapping a snack called *miang kham* in Thailand (Figure 1H). Previous studies suggested that plant leaves as a wrapping material could extend the storage duration of foods [16,18], while several of these leaves possess useful biological activities such as antioxidant and antibacterial activities [18]. 

There is evidence showing that factors that contribute to the spoilage of food are oxidation and microbial contamination [1]. For instance, the oxidation of lipids, especially unsaturated lipids, by atmospheric oxygen leads to changes in the lipid molecular structure to hydroperoxide and other free radicals [19]. The final products of the oxidation process continuously facilitate protein oxidation, resulting in protein carbonylation, polymerization, and coagulation [20]. These changes lead to the deterioration of the odor, taste, texture, and nutritional value of foods. Thus, in this study, plant leaves that have been used as food packaging were investigated for their antioxidant and antimicrobial activities. Selected plants, namely *N. nucifera* (**1**), *C. nucifera* (**2**), *N. fruticans* (**3**), *N. mirabilis* (**4**), *D. asper* (**5**), *C. pergracile* (**6**), *M. balbisiana* (**7**), and *P. sarmentosum* (**8**) (Table 1), were investigated in terms of their chemical profile, antioxidant compounds, and antioxidation capacity. To classify the types of chemical components, the HPTLC plate of extracts was sprayed with the NP reagent to show different fluorescent colors depending on the type of phenolic compounds. The NP-sprayed HPTLC plate of extracts **1**, **3**, **4**, **6**, and **7** showed yellow and orange bands (Figure 2A), representing flavonoids and flavonoid glycosides, e.g., hyperoside, isoquercitrin, luteolin, luteolin 7-O-glucoside, rutin, quercetin, quercitrin, and vitexin [21], while extracts **2**, **3** and **6** showed light blue spots (Figure 2A), pointing to the presence of phenolics, e.g., caffeic acid and chlorogenic acid [21]. The DPPH-sprayed HPTLC plate of leaf extracts **1**–**4** displayed far more yellow spots than that of extracts **5**–**8** (Figure 2B), in both number and intensity, suggesting that extracts **1**–**4** may have stronger antioxidant activity than that of extracts **5**–**8**. These yellow spots indicated the presence of antioxidant components, which were possibly flavonoids, saponins, or phenolic compounds [22]. The overlapping bands between the DPPH- and NP-sprayed HPTLC plates, notably observed for extracts **1**–**4**, confirmed the antioxidant activity of several flavonoid and phenolic compounds. Since phenolic compounds are usually associated with antioxidant and antimicrobial properties [23], the total phenolic content of extracts **1**–**8** was further evaluated in parallel with their antioxidant activities (Table 2). The FC assay, a method for the determination of phenolic content, is used to measure the antioxidant capacity of samples through the reduction of Mo^6+^ to Mo^5+^ [24]. The FC assay is quite rapid and reproducible and can be used to show a correlation between antioxidant activity and total phenolic content [25]. However, the FC assay is sensitive to not only phenolics but also other reducing compounds, i.e., reducing sugar and ascorbic acid, leading to biased FC results [26]. Due to this limitation, different types of antioxidation assays were needed. The DPPH assay is typically used to evaluate antioxidation activity through the reduction of 2,2-diphenyl-1-picrylhydrazyl radical [27]. The reduction mechanism of DPPH could be either a single electron transfer (SET) or hydrogen atom transfer (HAT) mechanism [24]. The performance of this method is limited by the reaction kinetics of DPPH, which depend on the type of antioxidants. Some antioxidants, such as ascorbic acid, react rapidly with DPPH, while some other antioxidants react slower or are even inert toward DPPH [28]. Moreover, the reaction of DPPH with some compounds is reversible, resulting in falsely low readings for antioxidant capacity [28]. Thus, a FRAP assay based on the reduction of Fe^3+^ to Fe^2+^ was performed [29]. However, if any compounds in the reaction have redox potentials lower than that of Fe^3+^ (0.70 V), then they can reduce Fe^3+^, leading to the underestimation of antioxidant activity [29]. Thus, the superoxide radical scavenging assay was applied in this study to evaluate antioxidant activity against superoxide (O_2_^•−^), which is produced from light-activated riboflavin [12]. Unlike DPPH, which is a synthetic radical, superoxide is a reactive radical species involved in lipid peroxidation [24]. Taken all together, to obtain reliable total antioxidant capacity results, various antioxidant assays with different mechanisms were conducted in parallel [28]. According to our study, extracts **1**–**4** were proven to display higher phenolic content and antioxidant activity than extracts **5**–**8**. There was also a direct correlation between the phenolic content and antioxidant activity of extracts **1**–**4**, which is consistent with the results revealed by other research groups [30,31]. The correlation between total phenolic content and antioxidant activity was also in agreement with the results of the DPPH- and NP-HPTLC screening of extracts **1**–**4**, which indicated that several types of phenolic compounds exhibited strong radical scavenging activity that reflected the ability of the leaf extracts to prevent or delay food spoilage. 

Microbial contamination is the main factor that leads to food spoilage and food poisoning [1]. Bacteria cause spoilage by consuming nutrients and moisture in foods, and most of them are also pathogens for humans. Some strains of Gram-positive *E. coli* produce enterotoxins and Shiga toxin, which cause diarrheal illness [32], dysentery, and hemolytic uremic syndrome [33]. *S. aureus* is a Gram-positive bacterium found in meat and poultry [1]. *S. aureus* infects humans and produces toxins that cause many diseases, from mild skin infections to severe pneumonia [34]. *Salmonella* species are Gram-negative bacteria responsible for salmonellosis, resulting in mild diarrhea to acute gastroenteritis [35]. *A. niger* is a type of mold that forms black colonies on spoiled foods. This microbe can secrete ochratoxin A, which is recognized as a nephrotoxin and a carcinogen [36]. 

According to the information above, extracts **1**–**8** were investigated for antimicrobial activity against *S. aureus, B. cereus, L. monocytogenes, E. coli, Salmonella* Abony, *C. albicans*, and *A. niger* (Figure 3). Interestingly, there was a relationship between the total phenolic content and antimicrobial activity of the extracts, as observed for extracts **1**–**4**, which displayed higher phenolic contents and higher antimicrobial efficacies than extracts **5**–**8** (Table 3). The correlation between antimicrobial activity and phenolic content was also observed by Nsor-Atindana et al. [37] and Jalal et al. [38], who reported the correlations between the total phenolic content and antimicrobial activity of *Theobroma cacao* and *Artocarpus altilis* extracts. Several studies suggested that the antimicrobial activity of phenolic compounds depends on the type of the phenolic compounds, the type of tested bacteria, including Gram-positive or Gram-negative bacteria, and the mechanisms of action. Phenolic compounds such as phenolic acids and flavonoids can damage and disrupt membrane functions, and inhibit bacteria enzymes, leading to bacteria cell death [39]. Thus, the phenolic content of the plant extracts could be the main factor contributing to antimicrobial activity. However, there are other chemical constituents that may also contribute to antimicrobial activity. Regarding extracts **1**–**4**, the antioxidant and microbial activities of *N. nucifera* (**1**), *C. nucifera* (**2**), and *N. fruticans* (**3**) extracts were reported by several research groups. In previous reports, *N. nucifera* leaf extract exhibited antioxidant activity observed in DPPH and ABTS assays [40], while the ethanolic extract of its flowers was proven to inhibit *S. aureus*, *P. aeruginosa*, and *C. albicans* [41]. Another research group highlighted the antioxidant and antimicrobial activities of *C. nucifera* leaf extract against *Acinetobacter* spp., *B. cereus*, *E. coli*, *S. dysenteriae*, *S. typhi*, and *A. niger* [42]. *N. fruticans* leaf extract was investigated to show antimicrobial activity against *S. aureus*, *E. coli*, *K. pneumonia*, *S. epidermidis*, and *P. aeruginosa* [43]. Although the *N. mirabilis* extract (**4**) exhibited the greatest antimicrobial activity in this study, the use of *N. mirabilis* as food packaging is exceptionally rare, compared with that of *N. fruticans*. Furthermore, the distribution, population usage, and biomass of *N. fruticans* are much greater than those of *N. mirabilis*, so the study of the biological activity and development of *N. fruticans* use is much more applicable. Thus, the bioassay-guided isolation of *N. fruticans* extract (**3**) was carried out to identify the bioactive compounds that are responsible for its antimicrobial activity. 

Based on the results of bioassay-guided fractionation, the EtOAc fraction exhibited the highest antimicrobial activity among the five fractions (Table 4 and Figure 4), followed by the water, BuOH, dichloromethane, and hexane fractions. The difference in the antimicrobial activity of each fraction could be attributed to the type and concentration of phenolic compounds, which may be most favored by the polarity of EtOAc and least favored by the polarity of hexanes. Using the results of antimicrobial analysis, the EtOAc fraction was further separated to obtain 3-*O*-caffeoyl shikimic acid (**I**), isoorientin (**II**), and isovitexin (**III**). Compound **I** has been found in many plants, such as *Phyllostachys pracecox* [44], which belongs to the Poaceae family, and *Phoenix dactylifera* [45], which belongs to the Arecaceae family as *N. fruticans*. A previous study proved that 3-*O*-caffeoyl shikimic acid possessed antioxidant activity [46]. Here, this is the first time that 3-*O*-caffeoyl shikimic acid was isolated from *N. fruticans* and identified for its antibacterial activity (Table 5). Isoorientin (**II**) is a flavonoid glycoside found in many plants, such as *Rhapis excelsa* in the Arecaceae family [47], *Stellaria nemorum*, and *Stellaria holostea* in the Caryophyllaceae family [48]. Previous studies also reported various biological activities of isoorientin, such as anti-inflammatory [49], antioxidant, and antibiotic activities [50]. Isovitexin (**III**) has been isolated from some plants such as the aerial part of *Lythrum salicaria* [51] and the leaves of *Gentiana* spp. [52]. In a previous study, isovitexin and isoorientin were proven to show antioxidant and antibacterial activities against various types of bacteria, including *S. aureus, E. faecalis, E. coli*, and *P. aeruginosa* [53]. In this study, 3-*O*-caffeoyl shikimic acid (**I**), isoorientin (**II**), and isovitexin (**III**) were proven to show antibacterial activity against five foodborne pathogens, suggesting that compounds **I**–**III** were responsible for the antimicrobial activity of *N. fruticans* ethanolic leaf extract. Even though all three bioactive components have been isolated from *N. fruticans* leaves, the bioactivity of *N. fruticans* crude extract is practically preferred to be used for food industrial applications rather than pure compounds. Thus, additional tests are required to study the antimicrobial activity of isolated compounds and crude extract. 

A limitation of our study is that not all of the bioactive components in the *N. fruticans* ethanolic extract were extensively identified. Some fractions were not subjected to isolate bioactive compounds due to their lesser bioactivity than that of the EtOAc fraction. However, those fractions still exhibited antimicrobial activity against some bacteria strains, suggesting that different compounds may contribute to the bioactivity of *N. fruticans* extract. Further studies are needed to identify compounds with antimicrobial activity against food spoilage and foodborne pathogens. Also, the retention of antimicrobial activities of the extracts or isolated compounds from the food packaging material over a period of time is worth investigating in the future.

## 5. Conclusions

In this study, the leaf ethanolic extracts of *N. nucifera*, *C. nucifera*, *N. fruticans*, and *N. mirabilis* displayed distinctive total phenolic contents, strong antioxidant activity, and potent antimicrobial activity against food spoilage microbes and food pathogens, namely *S. aureus*, *B. cereus*, *L. monocytogenes*, and *E. coli*. The results support the traditional use of *N. nucifera*, *C. nucifera*, *N. fruticans*, and *N. mirabilis* as natural food packaging, which can maintain the freshness of foods. In addition, 3-*O*-caffeoyl shikimic acid (**I**), isoorientin (**II**), and isovitexin (**III**) were isolated from *N. fruticans* leaf extract and tested in terms of their antimicrobial activity for the first time. This study demonstrates that the biological activity of leaves that are used in traditional food packaging helps retard food spoilage. The selected plants and their chemical constituents could be developed into biofilm packaging and natural food preservatives to maintain food quality, ensure food safety, and prolong the shelf life of food products. This study also promotes the traditional use of plants and adds value to these plants as natural packaging resources.

## Figures and Tables

**Table 1:** The leaf samples used in this study with their family, voucher no., places of collection, and extraction yield.

Plant Species	Family	Voucher No.	Places of Collection	Extraction Yield (%)
*Nelumbo nucifera* Gaertn. (**1**)	Nelumbonaceae	SS-856	Chiang Mai	39.52
*Cocos nucifera* L. (**2**)	Arecaceae	SS-857	Nakhon Ratchasima	19.91
*Nypa fruticans* Wurmb. (**3**)	Arecaceae	SS-858	Nakhon Ratchasima	14.15
*Nepenthes mirabilis* (Lour.) Druce (**4**)	Nepenthaceae	SS-859	Chiang Mai	17.47
*Dendrocalamus asper* (Schult & Schult.f.) Backer (**5**)	Poaceae	SS-860	Nakhon Ratchasima	11.41
*Cephalostachyum pergracile* Munro (**6**)	Poaceae	SS-861	Nakhon Ratchasima	7.93
*Musa balbisiana* Colla. (**7**)	Musaceae	SS-862	Bangkok	5.20
*Piper sarmentosum* Roxb. (**8**)	Piperaceae	SS-863	Bangkok	17.08

**Table 2:** Antioxidant activity of selected leaf extracts, including total phenolic content, DPPH radical scavenging activity, ferric-reducing antioxidant power capability, and superoxide radical scavenging activity.

Leaf Extract	TPC (mg GAE/g)	DPPH (IC_50_, μg/mL)	FRAP (μmol Fe^2+^/g)	SRSA (IC_50_, μg/mL)
*N. nucifera* (**1**)	115.15 ± 3.84 ^a^	14.71 ± 0.56 ^f^	545.72 ± 10.80 ^a^	11.19 ± 0.63 ^f^
*C. nucifera* (**2**)	82.18 ± 4.03 ^c^	34.28 ± 0.55 ^e^	342.92 ± 8.51 ^b^	38.97 ± 1.05 ^c^
*N. fruticans* (**3**)	97.54 ± 2.06 ^b^	16.67 ± 0.55 ^f^	529.36 ± 5.44 ^a^	27.89 ± 1.84 ^d^
*N. mirabilis* (**4**)	108.39 ± 6.48 ^a^	14.93 ± 0.40 ^f^	551.38 ± 4.11 ^a^	20.16 ± 1.43 ^e^
*D. asper* (**5**)	34.43 ± 0.27 ^e^	142.16 ± 3.25 ^a^	54.57 ± 2.80 ^f^	>120 ^a^
*C. pergracile* (**6**)	41.03 ± 2.51 ^de^	61.69 ± 2.45 ^c^	104.60 ± 4.12 ^d^	>120 ^a^
*M. balbisiana* (**7**)	50.08 ± 2.32 ^d^	46.70 ± 1.08 ^d^	191.78 ± 2.68 ^c^	>120 ^a^
*P. sarmentosum* (**8**)	38.58 ± 1.73 ^e^	116.09 ± 2.89 ^b^	98.69 ± 2.54 ^d^	69.05 ± 1.50 ^b^
Ascorbic acid ^1^	nt	2.27 ± 0.20	1371.60 ± 21.93	nt
Quercetin ^2^	nt	nt	nt	5.50 ± 0.45
Trolox ^2^	nt	nt	nt	57.34 ± 1.90

TPC: total phenolic content; DPPH: DPPH scavenging activity; FRAP: ferric-reducing antioxidant power; SRSA: superoxide radical scavenging activity. ^1^ Ascorbic acid was used as a positive control in DPPH and FRAP assays. ^2^ Quercetin and Trolox were used as positive controls for the SRSA assay. Values with the same superscript letter within a column are not significantly different at *p* < 0.05; (nt): not tested.

**Table 3:** The inhibition zones of selected leaf extracts against food spoilage microbes and foodborne pathogens.

Leaf Extract	Inhibition Zone Diameter (mm) ± SD						
	*S. aureus*	*B. cereus*	*L. monocytogenes*	*E. coli*	*Salmonella* Abony	*C. albicans*	*A. niger*
*N. nucifera* (**1**)	10.47 ± 0.58 ^b^	10.70 ± 1.30 ^b^	10.60 ± 0.17 ^a^	12.47 ± 0.21 ^b^	-	-	-
*C. nucifera* (**2**)	8.55 ± 0.21 ^c^	10.17 ± 0.90 ^b^	7.75 ± 0.92 ^b^	10.13 ± 0.47 ^c^	-	-	-
*N. fruticans* (**3**)	9.23 ± 0.47 ^c^	10.67 ± 0.21 ^b^	8.87 ± 0.42 ^b^	9.85 ± 0.78 ^c^	-	-	-
*N. mirabilis* (**4**)	17.77 ± 0.15 ^a^	13.10 ± 0.70 ^a^	10.93 ± 0.61 ^a^	10.77 ± 0.12 ^c^	8.70 ± 0.53	24.10 ± 0.46	-
*D. asper* (**5**)	-	7.80 ± 0.40 ^c^	-	14.67 ± 0.21 ^a^	-	-	-
*C. pergracile* (**6**)	-	-	-	7.73 ± 0.12 ^d^	-	-	-
*M. balbisiana* (**7**)	-	-	-	7.33 ± 0.35 ^d^	-	-	-
*P. sarmentosum* (**8**)	-	-	-	7.20 ± 0.17 ^d^	-	-	-
Vehicle control	0.00 ± 0.00	0.00 ± 0.00	0.00 ± 0.00	0.00 ± 0.00	0.00 ± 0.00	0.00 ± 0.00	0.00 ± 0.00
Amphotericin B	nt	nt	nt	nt	nt	19.83 ± 0.21	10.67 ± 0.25
Gentamycin	26.47 ± 0.12	24.47 ± 0.15	24.70 ± 0.20	25.37 ± 0.15	nt	nt	nt

The concentration of leaf extracts was 10 mg/disc; DMSO was used as a vehicle control; gentamycin and amphotericin B (10 µg/disc) were used as positive controls. Values with the same superscript letter within a column are not significantly different at *p* < 0.05; (-): no inhibition zone; (nt): not tested.

**Table 4:** Inhibition zones of *N. fruticans* extract **(3)** and its different solvent fractions against food spoilage microbes and foodborne pathogens.

Extract/Fraction	Inhibition Zone Diameter (mm) ± SD						
	*S. aureus*	*B. cereus*	*L. monocytogenes*	*E. coli*	*Salmonella* Abony	*C. albicans*	*A. niger*
**Extract**							
*N. fruticans* **(3)**	13.00 ± 0.00 ^cd^	12.55 ± 0.35 ^bc^	9.10 ± 0.26 ^bc^	11.10 ± 0.57 ^bc^	-	-	-
**Fraction**							
Hexanes **(F1)**	8.93 ± 0.35 ^e^	8.27 ±0.12 ^d^	8.53 ± 0.15 ^c^	-	-	-	-
Dichloromethane **(F2)**	11.13 ± 1.81 ^de^	10.37 ± 1.57 ^cd^	7.83 ± 0.85 ^c^	8.07 ± 0.51 ^d^	-	-	-
Ethyl acetate **(F3)**	17.33 ± 0.35 ^a^	17.27 ± 0.55 ^a^	14.03 ± 0.55 ^a^	14.27 ± 0.49 ^a^	9.67 ± 0.12	-	-
Butanol **(F4)**	14.00 ± 0.00 ^bc^	11.73 ± 1.43 ^bc^	8.83 ± 0.61 ^c^	10.20 ± 1.45 ^c^	-	-	-
Water **(F5)**	15.67 ± 0.86 ^ab^	13.20 ± 0.44 ^b^	10.27 ± 0.25 ^b^	12.20 ± 0.46 ^b^	-	-	-
Vehicle control	0.00 ± 0.00	0.00 ± 0.00	0.00 ± 0.00	0.00 ± 0.00	0.00 ± 0.00	0.00 ± 0.00	0.00 ± 0.00

The concentration of *N. fruticans* extract and fractions were 10 mg/disc; DMSO was used as a vehicle control. Values with the same superscript letter within a column are not significantly different at *p* < 0.05; (-): no inhibition zone; (nt): not tested.

**Table 5:** Minimum inhibitory concentration (MIC) of three compounds isolated from *N. fruticans* against five foodborne pathogens.

Compound	Minimum Inhibitory Concentration (µg/mL)				
	*S. aureus*	*B. cereus*	*L. monocytogenes*	*E. coli*	*Salmonella* Abony
3-*O*-Caffeoyl shikimic acid (**I**)	1000	800	1000	1000	1000
Isoorientin (**II**)	1000	1000	1000	1000	1000
Isovitexin (**III**)	>1000	800	1000	800	>1000
Gentamycin	0.47	1.90	0.12	1.90	7.59

Gentamycin was used as a positive control.

## Data Availability

Data is contained within the article or supplementary material.

## Supplementary Materials

The following supporting information can be downloaded at: https://www.mdpi.com/article/10.3390/foods12122409/s1: Table S1. ^1^H and ^13^C NMR signals of compound **I** in comparison with those from a previous report. Table S2. ^1^H and ^13^C NMR signals of compound **II** in comparison with those from a previous report. Table S3. ^1^H and ^13^C NMR signals of compound **III** in comparison with those from a previous report. Figure S1. ^1^H NMR spectrum of compound **I** in DMSO**.** Figure S2. ^13^C NMR spectrum of compound **I** in DMSO. Figure S3. ^1^H NMR spectrum of compound **II** in DMSO. Figure S4. ^13^C NMR spectrum of compound **II** in DMSO. Figure S5. DEPT-135 spectrum of compound **II** in DMSO. Figure S6. ^1^H NMR spectrum of compound **III** in DMSO. Figure S7. ^13^C NMR spectrum of compound **III** in DMSO.
