# Supplementary material for: Antimicrobial Activity against Foodborne Pathogens and Antioxidant Activity of Plant Leaves Traditionally Used as Food Packaging
Source: Foods. 2023 Jun 19;12(12):2409. doi: 10.3390/foods12122409 (PMC10297150; doi:10.3390/foods12122409)
Supplement: Supplementary file 1 [file foods-12-02409-s001.zip › foods-2410532-supplementary.pdf]

Supplementary material

Table S1. ^{1}H and ^{13}C NMR signals of compound **I** in comparison with those from a previous report.

Position	Compound I[a]		3-*O*-caffeoylshikimic acid[a,b]	
	δ_H (multiplicity, J_{HH} in Hz)	δ_C	δ_H (multiplicity, J_{HH} in Hz)	δ_C
1		125.4		125.9
2	7.03 (1H, d, 1.9)	114.8	7.07 (1H, d, 1.5)	115.4
3		145.5		146.1
4		148.4		149.0
5	6.74 (1H, d, 7.8)	115.7	6.77 (1H, d, 8.3)	116.3
6	7.00 (1H, d, 7.8, 1.9)	121.3	7.01 (1H, dd, 8.3, 1.5)	121.9
7	7.46 (1H, d, 15.0)	145.3	7.48 (1H, d, 16,0)	146.0
8	6.23 (1H, d, 15.0)	114.4	6.25 (1H, d, 16.0)	144.4
9		166.1		166.6
1'		128.2		128.9
2'	6.67 (1H, m)	138.6	6.68 (1H, m)	138.7
3'	5.02 (1H, m)	69.8	5.09 (1H, m)	70.4
4'	3.74 (1H, m)	67.7	3.76 (1H, m)	68.3
5'	4.25 (1H, brs)	65.4	4.26 (1H, brs)	65.9
6'	2.17 (1H, dd, 17.5, 4.2)	27.7	2.18 (1H, dd, 18.0, 4.3)	28.3
	2.64 (1H, brd, 17.5)		2.63 (1H, dd, 18.0, 2.5)	
7'		167.4		168.2

[a]DMSO-*d*6 was used as a solvent.

[b]Li, X.; Zhang, Y.; Zeng, X.; Yang, L.; Deng, Y. Chemical profiling of bioactive constituents in *Sarcandra glabra* and its preparations using ultra-high-pressure liquid chromatography coupled with LTQ Orbitrap mass spectrometry. *Rapid Commun. Mass Spectrom.* **2011**, 25, 2439–2447.

Table S2. ^{1}H and ^{13}C NMR signals of compound **II** in comparison with those from a previous report.

Position	Compound II[a]		Isoorientin[a,b]	
	δ_H (multiplicity, J_{HH} in Hz)	δ_C	δ_H (multiplicity, J_{HH} in Hz)	δ_C
2		163.7		163.7
3	6.67 (1H, s)	103.5	6.66 (1H, s)	102.9
4		181.9		181.9
5		160.7		160.8
6		108.4		108.9
7		163.4		163.4
8	6.48 (1H, s)	93.3	6.47 (1H, s)	93.6
9		156.2		156.3
10		103.8		103.5
1'		121.6		121.5
2'	7.39 (1H, s)	113.1	7.39 (1H, d, 2.2)	113.4
3'		145.8		145.8
4'		149.7		149.8
5'	6.87 (1H, d, 8.1)	115.9	6.88 (1H, d, 8.4)	116.1
6'	7.40 (1H, d, 8.1)	118.8	7.41 (1H, dd, 8.4, 2.2)	119.0
1''	4.57 (1H, d, 9.8)	72.8	4.57 (1H, d, 9.7)	73.1
2''	4.03 (1H, t, 9.4)	70.4	4.05 (1H, t, 9.4)	70.3
3''	3.19 (1H, m)	78.8	3.19 (1H, t, 8.6)	79.0
4''	3.14 (1H, m)	70.0	3.11 (1H, t, 9.2)	70.7
5''	3.15 (1H, m)	81.4	3.15, (1H, m)	81.7
6''	3.67 (1H, m)	61.3	3.68 (1H, dd, 12.0, 1.8)	61.6
	3.40 (1H, m)		3.40 (1H, dd, 12.0, 6.3)	

[a]DMSO-*d*6 was used as a solvent.

[b] Rayyan, S.; Fossen, T.; Solheim Nateland, H.; Andersen, O.M. Isolation and Identification of Flavonoids, Including Flavone Rotamers, From The Herbal Drug 'Crataegi Folium Cum Flore' (Hawthorn). *Phytochem. Anal.* **2005**, 16, 334–341.

Table S3. ^{1}H and ^{13}C NMR signals of compound **III** in comparison with those from a previous report.

Position	Compound 3[a]		Isovitexin[a,b]	
	δ_H (multiplicity, J_{HH} in Hz)	δ_C	δ_H (multiplicity, J_{HH} in Hz)	δ_C
2		163.5		163.4
3	6.79 (1H, s)	102.8	6.77 (1H, s)	102.6
4		182.0		181.8
5		161.2		161.0
6		108.9		108.7
7		163.2		163.2
8	6.50 (1H, s)	93.6	6.51 (1H, s)	93.5
9		156.2		156.0
10		103.4		103.2
1'		121.1		121.0
2'	7.92 (1H, d, 8.8)	128.5	7.92 (1H, d, 8.7)	128.3
3'	6.92 (1H, d, 8.8)	116.1	6.93 (1H, d, 8.7)	115.9
4'		160.7		160.5
5'	6.92 (1H, d, 8.8)	116.0	6.93 (1H, d, 8.7)	115.9
6'	7.92 (1H, d, 8.8)	128.6	7.92 (1H, d, 8.7)	128.3
1''	4.59 (1H, d, 9.8)	73.1	4.59 (1H, d, 9.8)	72.9
2''	4.07 (1H, m)	70.6	4.04 (1H, t, 9.1)	70.5
3''	3.20 (1H, m)	78.9	3.21 (1H, t, 8.4)	78.8
4''	3.14 (1H, m)	70.2	3.13 (1H, d, 9.2)	70.1
5''	3.16 (1H, m)	81.6	3.16, (1H, m)	81.4
6''	3.67 (1H, m)	61.5	3.61 (1H, brd, 10.9)	61.3
	3.40 (1H, m)		3.40 (1H, m)	

[a]DMSO-*d*6 was used as a solvent.

[b]Jayasinghe, U.L.B.; Valasooriya, B.A.I.S.; Bandara, A.G.D.; Fujimoto, Y. Glycosides from *Grewia damine* and *Filicium decipiens*. *Nat. Prod. Res.* **2004**, 18(6), 499–502.

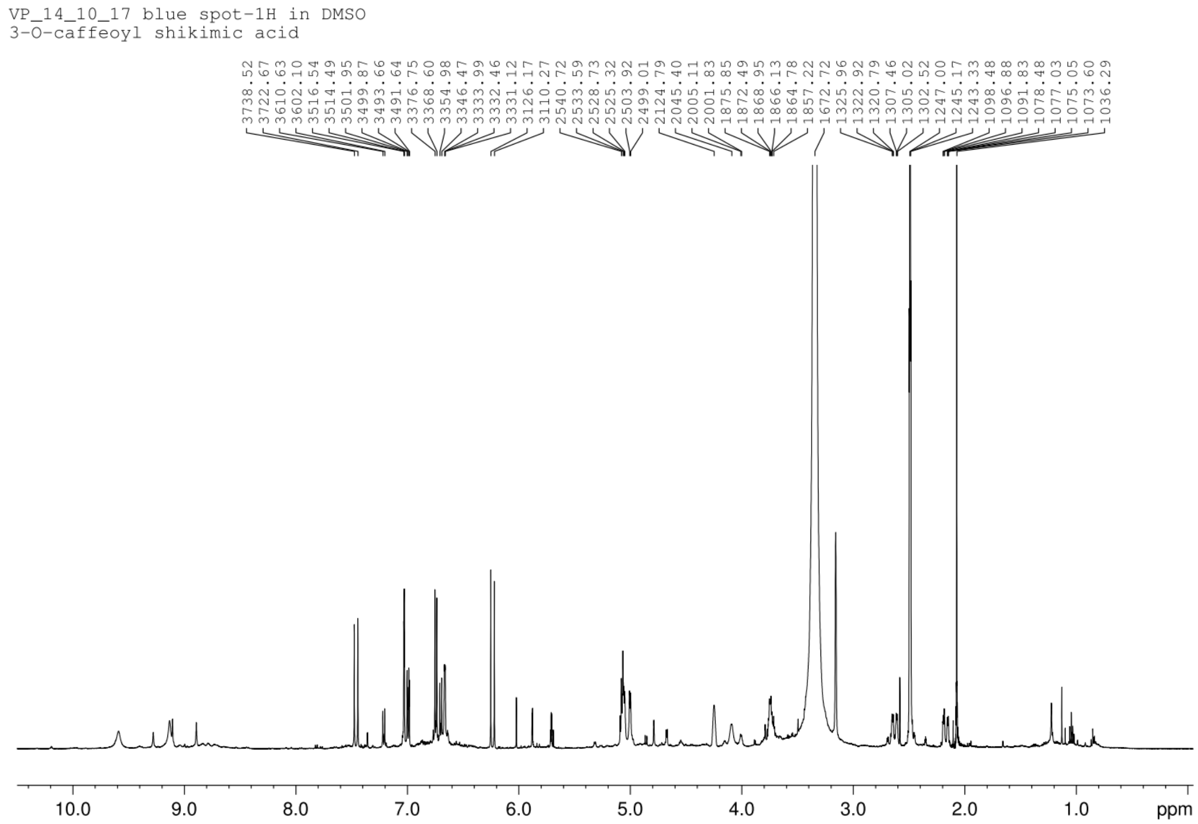

Figure S1. ^{1}H NMR spectrum of compound **I** in DMSO.

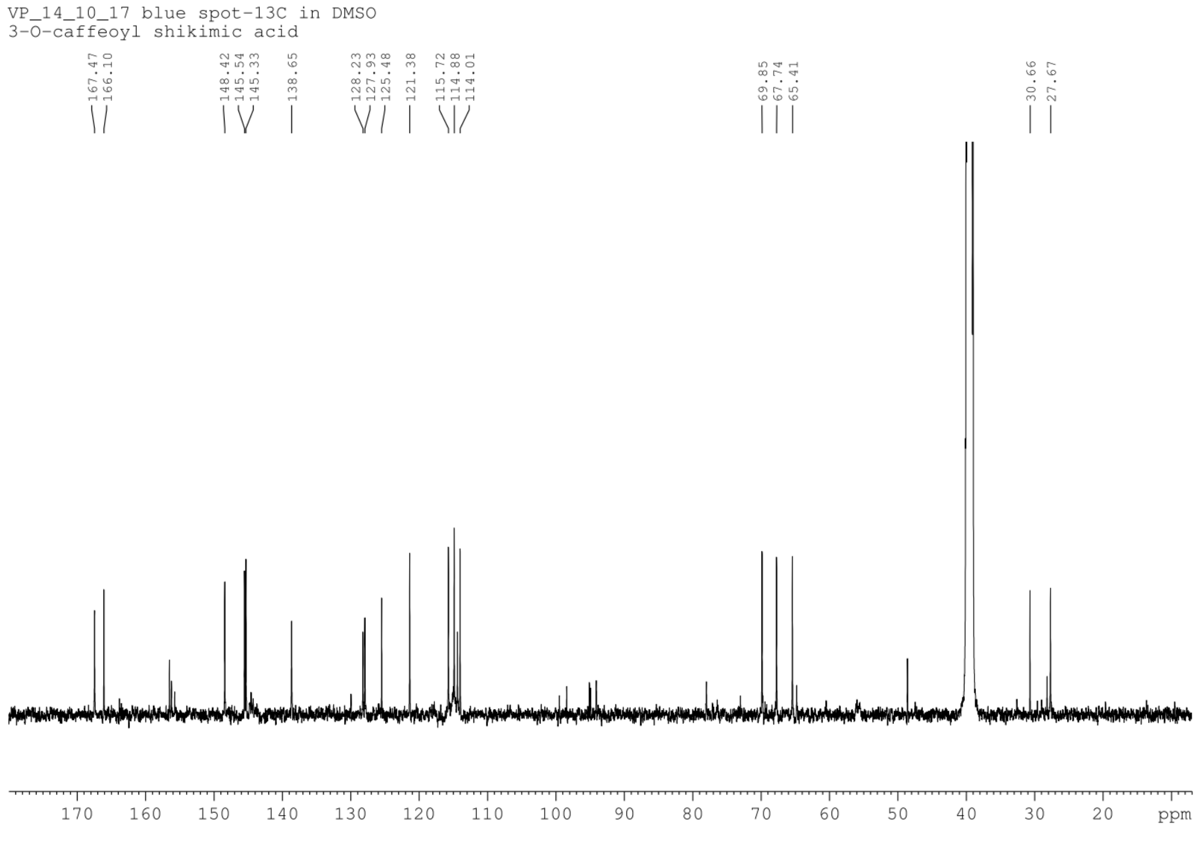

Figure S2. ^{13}C NMR spectrum of compound **I** in DMSO.

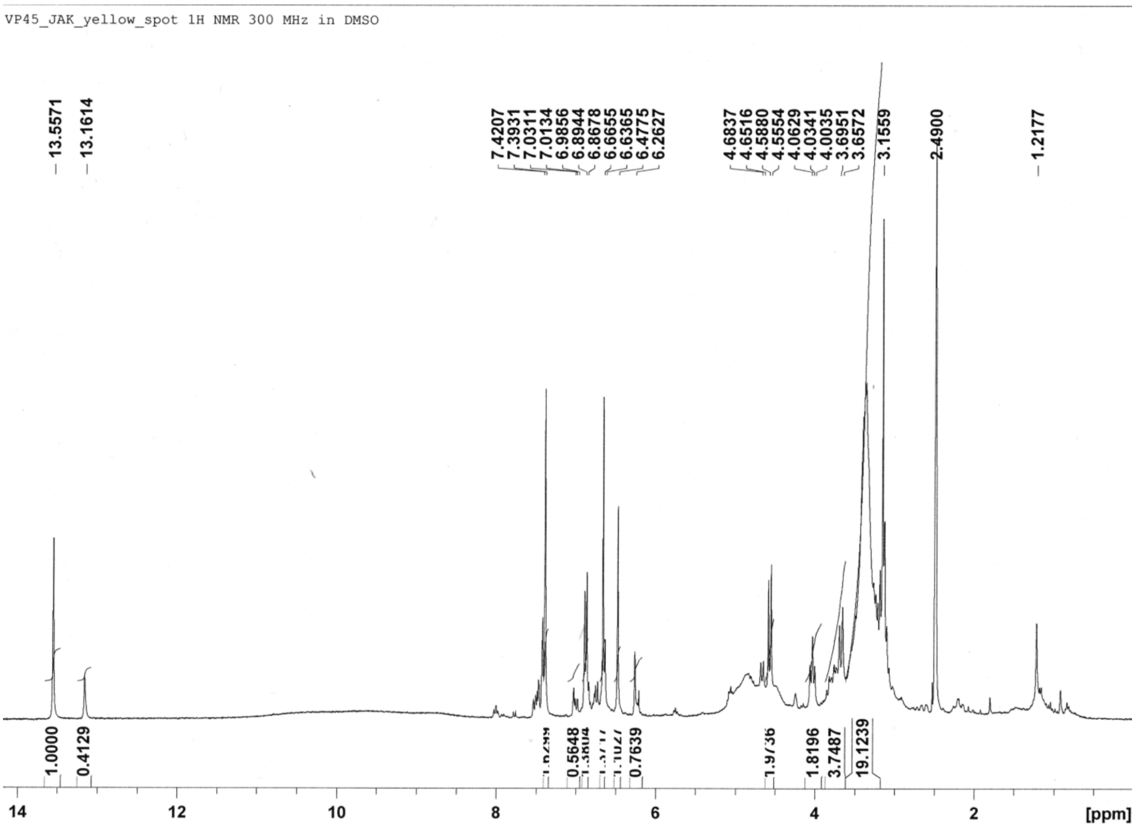

Figure S3. ^{1}H NMR spectrum of compound **II** in DMSO.

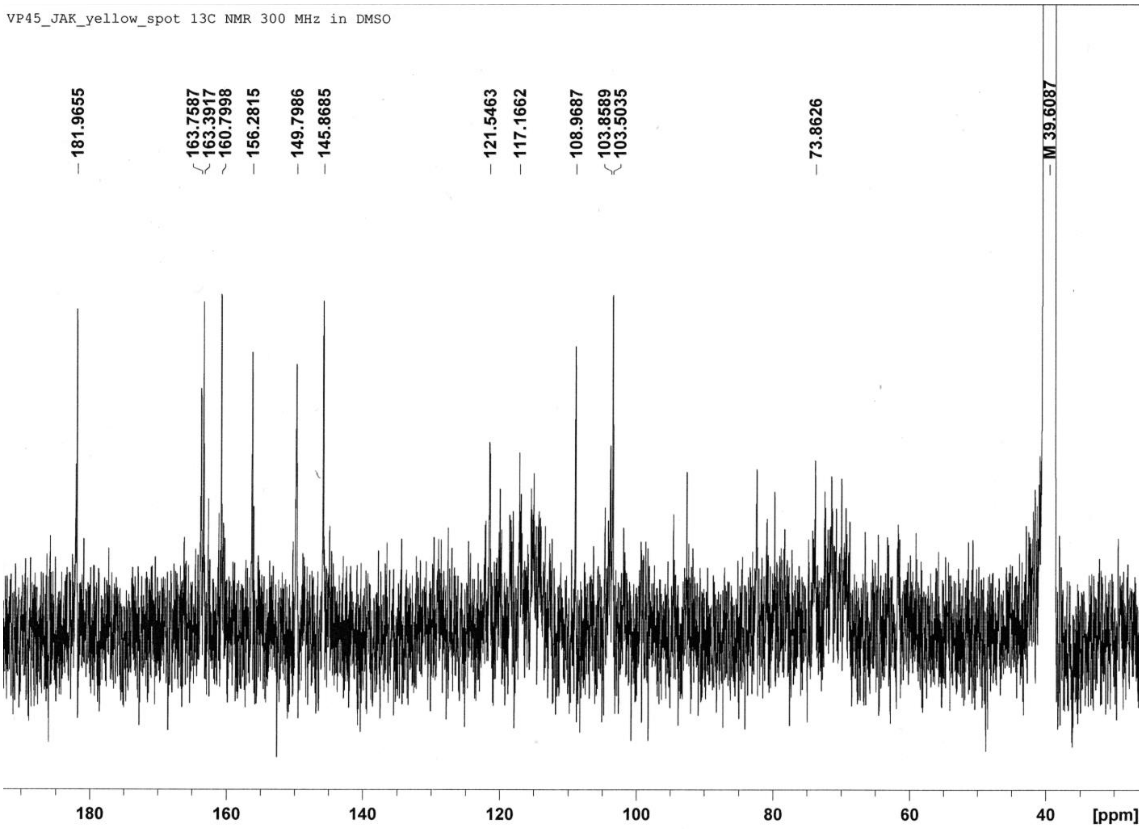

Figure S4. ^{13}C NMR spectrum of compound **II** in DMSO.

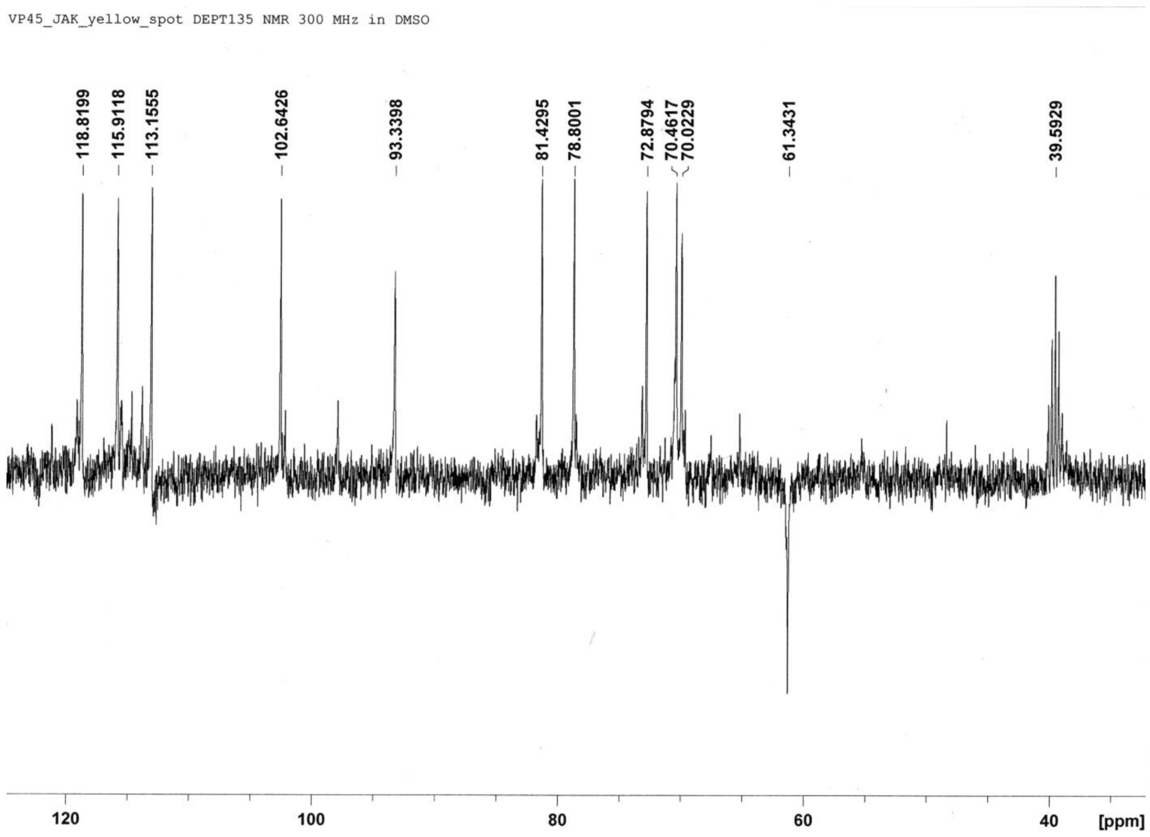

Figure S5. DEPT-135 spectrum of compound **II** in DMSO.

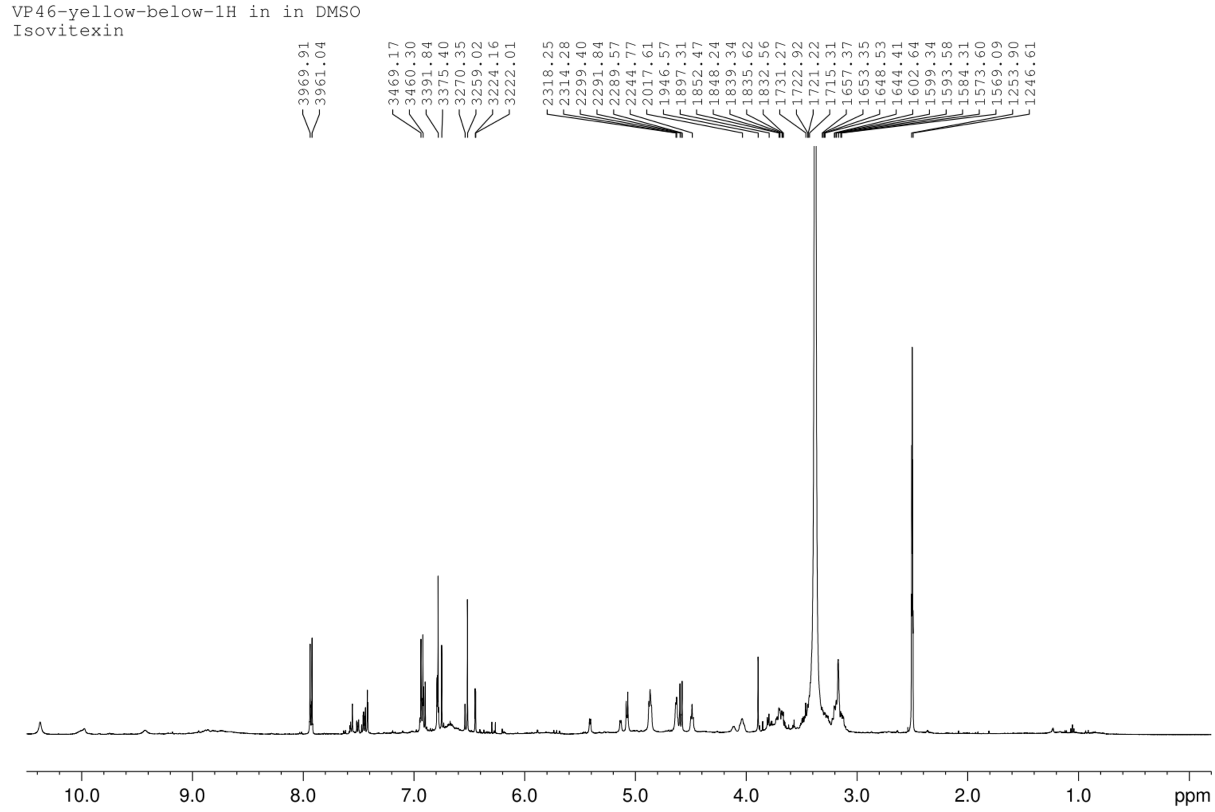

Figure S6. ^{1}H NMR spectrum of compound **III** in DMSO.

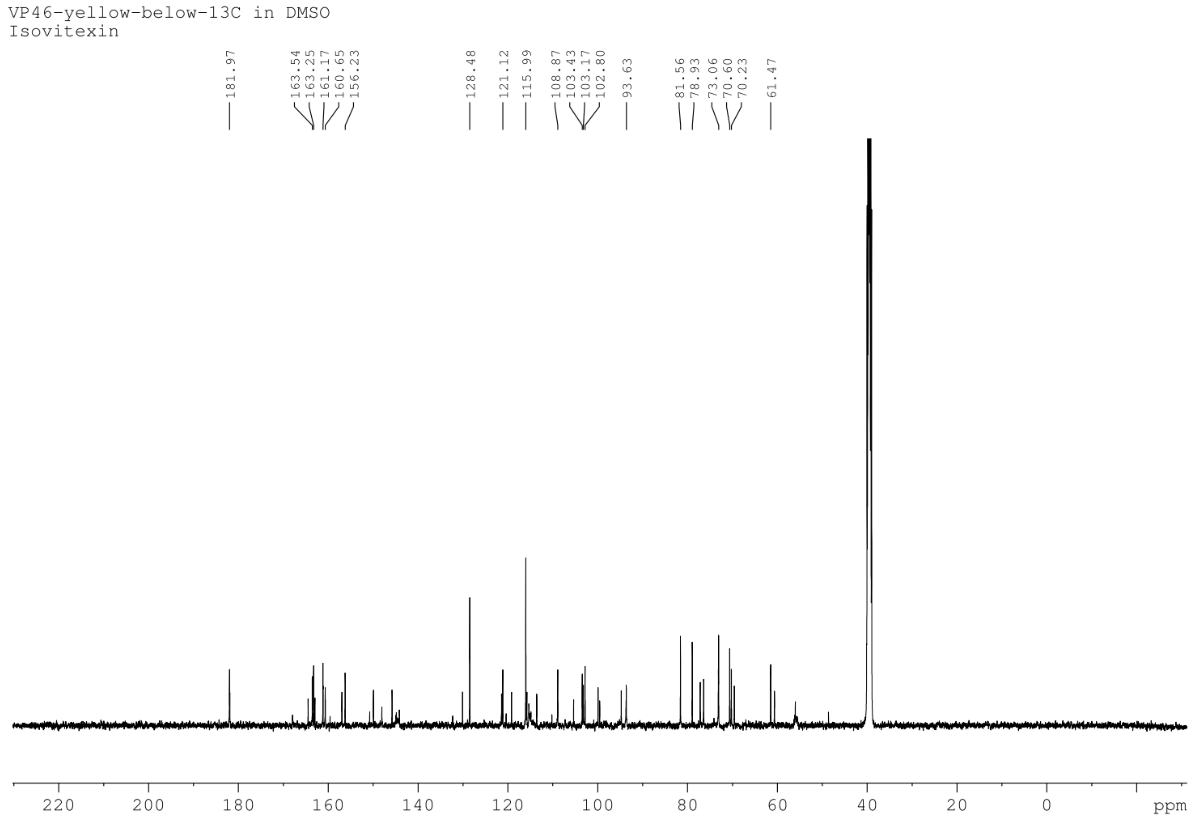

Figure S7. ^{13}C NMR spectrum of compound **III** in DMSO.
